# *Actinobaculum schaalii*: An Emerging Uropathogen?

**DOI:** 10.1155/2012/468516

**Published:** 2012-04-09

**Authors:** Peyman Tavassoli, Ryan Paterson, Jennifer Grant

**Affiliations:** ^1^Department of Pathology and Laboratory Medicine, University of British Columbia, Vancouver, BC, Canada V6T 2B5; ^2^Department of Urologic Sciences, University of British Columbia, Vancouver, BC, Canada V5Z 1M9

## Abstract

*A. schaalii* is a rare uropathogen. We report urosepsis with *Actinobaculum schaalii* detected serendipitously in blood and urine culture in a 79-year-old with urinary tract obstruction. This paper illuminates the flaws in our current system in detecting *A. schaalii* and raises awareness among clinicians and laboratory teams.

## 1. Introduction

*Actinobaculum schaalii* is a small Gram-positive coccoid rod that requires CO_2_ for optimal growth. Also in the genus *Actinobaculum* are *A. suis*, *A. urinale*, and *A. massiliense*. *A. suis* is known to cause urinary tract infections (UTIs) in swine [1, 2] and human UTIs have been reported with *A. urinale* and *A*. *massiliense* [3–5]. *A. schaalii*, which is the focus of this paper, was first described as a cause of human UTIs in 1997, with subsequent reports [6–8]. Growing evidence suggests that *A. schaalii* may be a more common uropathogen in the elderly than previously reported, especially those with obstructive uropathy [7–13].

Most laboratories incubate urine cultures at 35°C in ambient air. Since *A. schaalii* grows slowly in aerobic conditions with no CO_2_ supplementation, this bacterium's isolation and identification from urine specimens by standard laboratory methods is rare. Most laboratories do not look specifically for *Actinobaculum* species. Even if *A. schaalii *grows, this organism may be overlooked either because of overgrowth of common uropathogens or the bacteria's resemblance to normal skin flora [7, 8, 11].

To address this problem, Bank et al. developed a TaqMan real-time quantitative PCR and assessed 252 clinical urine samples [9]. They found 22% of urine samples were positive for *A. schaalii *in the over 60-year-old patient population, whilst only 7% of samples were positive in the less than 60-year-old cohort [9]. Using the same diagnostic technique in a cohort of patients with kidney stones, they showed that more than 24% of urine samples were positive for *A. schaalii*, which was the only pathogen in more than 60% of those patients [13].

## 2. Case Report

A 79-year-old man with a previous history of nephrolithiasis and benign prostatic hyperplasia was admitted with fever, chills, nausea, vomiting, and diarrhea. On presentation, he had an elevated leukocyte count of 17.2 × 10^3^ cells/*μ*L (90% neutrophils) and an elevated creatinine and lactate (155 *μ*mol/L and 2.6 mmol/L, resp.). His initial urinalysis tested positive for blood and leukocytes. An ultrasound of the left kidney showed left hydronephrosis and an obstructing stone in the left ureter. Empiric parenteral therapy with piperacillin-tazobactam was initiated and subsequently a left nephrostomy tube was placed. However, his condition deteriorated and the patient was transferred to the intensive care unit (ICU). A repeat urinalysis revealed 4+ polymorphonuclear cells, 4+ Gram-positive bacilli and was negative for nitrate. The initial urine culture was negative; however, an astute laboratory technologist noticed the discrepancy between the Gram-stain and the culture results. In consultation with the microbiologist, the technologist repeated the urine culture, including an anaerobic incubation. On repeat culture, the patient's urine culture grew 10 million CFU/L of a possible anaerobic Gram-positive bacillus with the blood culture also growing anaerobic Gram-positive bacilli. Due to the unusual nature of the cultures in this urosepsis patient, the samples were sent to the reference laboratory for microorganism identification. *A. schaalii *was identified from blood culture. Although the care providers were informed upon initial Gram-positive bacilli observation on smear, the final bacterial identification result was released 12 days after specimen receipt. Fortunately, *A. schaalii* is usually sensitive to pip-tazo and the patient became stable following a short stay in ICU. Subsequently, the patient's stone was unsuccessfully treated with extracorporeal shock wave lithotripsy (ESWL) and later definitively treated with an ureteroscopy and laser lithotripsy.

## 3. Discussion

*A. schaalii *is an emerging uropathogen mainly in elderly patients or patients with underlying urologic disease [7, 9, 13]. Although there are a few reports of nonurological infections [14, 15], the spectrum of infection primarily ranges from benign cystitis to severe pyelonephritis with urosepsis.* A. schaalii *has been reported in both inpatient and outpatient populations, either as an isolated organism or associated with other common uropathogens [8–11, 13]. Unfortunately, our routine laboratory methods will not identify *A. schaalii *from urine specimens as this bacterium requires CO_2_ and has sluggish growth in comparison to common uropathogens. For this reason, *A. schaalii *is identified from the blood rather than from the urine in 30% of identified cases [11].

Urologists are recommended to notify the laboratory if they are concerned about urinary infection with an unusual organism such as *A. schaalii*, based on risk factors or conflicting laboratory results. Accordingly, conventional laboratory methods must be modified to isolate *A. schaalii *from specimens upon such requests. In addition, laboratories should also consider *A. schaalii* as an etiology for UTI once there is no growth from the urine specimen, despite the presence of Gram-positive coccoid rods and/or leukocytes present on the urinalysis.

Another important issue relates to the susceptibility profile of *A. schaalii* is that it retains susceptibility to penicillins, 3rd generation cephalosporins, aminoglycosides, and nitrofurantoin. However, this organism is mainly resistant to ciprofloxacin and trimethoprim/sulfamethoxazole [11, 12], two commonly employed antibiotics in urology patients. To our knowledge, all of the *A. schaalii *reports and studies have been carried out in Europe with the exception of a single case report from North America [16]. Therefore, most of our clinicians are not aware of this potential uropathogen. We believe that a well-designed multicenter study is required to find out the prevalence and incidence of *A. schaalii*.

## References

[1] Walker RL, MacLachlan NJ (1989). Isolation of *Eubacterium suis* from sows with cystitis. *Journal of the American Veterinary Medical Association*.

[2] Yamini B, Slocombe RF (1988). Porcine abortion caused by *Actinomyces suis*. *Veterinary Pathology*.

[3] Greub G, Raoult D (2002). *Actinobaculum massiliae*, a new species causing chronic urinary tract infection. *Journal of Clinical Microbiology*.

[4] Fendukly F, Osterman B (2005). Isolation of *Actinobaculum schaalii* and *Actinobaculum urinale* from a patient with chronic renal failure. *Journal of Clinical Microbiology*.

[5] Hall V, Collins MD, Hutson RA, Falsen E, Inganäs E, Duerden BI (2003). *Actinobaculum urinale* sp. nov., from human urine. *International Journal of Systematic and Evolutionary Microbiology*.

[6] Lawson PA, Falsen E, Åkervall E, Vandamme P, Collins MD (1997). Characterization of some *Actinomyces*-like isolates from human clinical specimens: reclassification of *Actinomyces suis* (Soltys and Spratling) as *Actinobaculum suis* comb. nov. and description of *Actinobaculum schaalii* sp. nov. *International Journal of Systematic Bacteriology*.

[7] Tschudin-Sutter S, Frei R, Weisser M, Goldenberger D, Widmer AF (2011). *Actinobaculum schaalii*—invasive pathogen or innocent bystander? A retrospective observational study. *BMC Infectious Diseases*.

[8] Reinhard M, Prag J, Kemp M (2005). Ten cases of *Actinobaculum schaalii* infection: clinical relevance, bacterial identification, and antibiotic susceptibility. *Journal of Clinical Microbiology*.

[9] Bank S, Jensen A, Hansen TM, Søby KM, Prag J (2010). *Actinobaculum schaalii*, a common uropathogen in elderly patients, Denmark. *Emerging Infectious Diseases*.

[10] Nielsen HL, Søby KM, Christensen JJ, Prag J (2010). *Actinobaculum schaalii*: a common cause of urinary tract infection in the elderly population. Bacteriological and clinical characteristics. *Scandinavian Journal of Infectious Diseases*.

[11] Beguelin C, Genne D, Vara A (2011). *Actinobaculum schaalii*: clinical observation of 20 cases. *Clinical Microbiology and Infection*.

[12] Cattoir V, Varca A, Greub G, Prod’hom G, Legrand P, Lienhard R (2010). In vitro susceptibility of *Actinobaculum schaalii* to 12 antimicrobial agents and molecular analysis of fluoroquinolone resistance. *Journal of Antimicrobial Chemotherapy*.

[13] Bank S, Hansen TM, By KM, Lund L, Prag JR (2011). *Actinobaculum schaalii* in urological patients, screened with real-time polymerase chain reaction. *Scandinavian Journal of Urology and Nephrology*.

[14] Haller P, Bruderer T, Schaeren S (2007). Vertebral osteomyelitis caused by *Actinobaculum schaalii*: a difficult-to-diagnose and potentially invasive uropathogen. *European Journal of Clinical Microbiology and Infectious Diseases*.

[15] Hoenigl M, Leitner E, Valentin T (2010). Endocarditis caused by *Actinobaculum schaalii*, Austria. *Emerging Infectious Diseases*.

[16] Larios OE, Bernard KA, Manickam K, Ng B, Alfa M, Ronald A (2010). First report of *Actinobaculum schaalii* urinary tract infection in North America. *Diagnostic Microbiology and Infectious Disease*.

